# Influence of cross-linking and retrograde flow on formation and dynamics of lamellipodium

**DOI:** 10.1371/journal.pone.0213810

**Published:** 2019-03-21

**Authors:** Asal Atakhani, Farshid Mohammad-Rafiee, Azam Gholami

**Affiliations:** 1 Department of Physics, Institute for Advanced Studies in Basic Sciences (IASBS), Zanjan 45137-66731, Iran; 2 Research Center for Basic Sciences & Modern Technologies (RBST), Institute for Advanced Studies in Basic Sciences (IASBS), Zanjan 45137-66731, Iran; 3 Max Planck Institute for Dynamics and Self-Organization, Göttingen, Germany; Bioinformatics Institute, SINGAPORE

## Abstract

The forces that arise from the actin cortex play a crucial role in determining the membrane deformation. These include protrusive forces due to actin polymerization, pulling forces due to transient attachment of actin filaments to the membrane, retrograde flow powered by contraction of actomyosin network, and adhesion to the extracellular matrix. Here we present a theoretical model for membrane deformation resulting from the feedback between the membrane shape and the forces acting on the membrane. We model the membrane as a series of beads connected by springs and determine the final steady-state shape of the membrane arising from the interplay between pushing/pulling forces of the actin network and the resisting membrane tension. We specifically investigate the effect of the gel dynamics on the spatio-temporal deformation of the membrane until a stable lamellipodium is formed. We show that the retrograde flow and the cross-linking velocity play an essential role in the final elongation of the membrane. Interestingly, in the simulations where motor-induced contractility is switched off, reduced retrograde flow results in an increase in the rate and amplitude of membrane protrusion. These simulations are consistent with experimental observations that report an enhancement in protrusion efficiency as myosin II molecular motors are inhibited.

## Introduction

**Fig 1:**
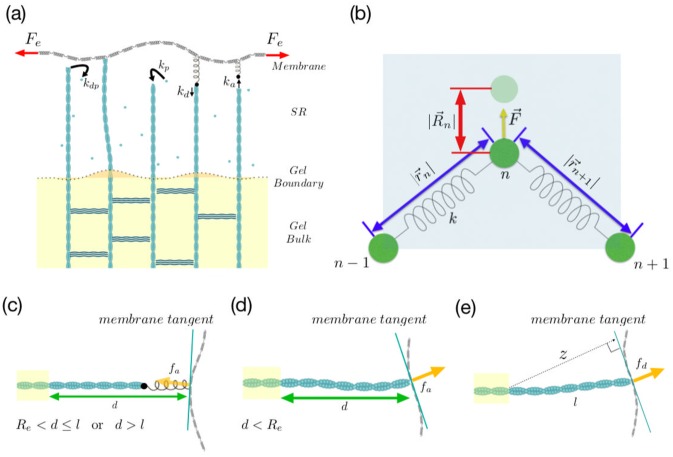
Model scheme. (a) Schematic representation of semiflexible region and actin gel in the model. (b) Magnification of three neighboring beads on the membrane plus the shaded area used for energy minimization. The faded filled circle represents the new position of the bead and defines the displacement vector $|{\vec{R}}_{n}|$. (c, d) Stretched and compressed configurations of an attached filament exerting pulling or pushing forces on the membrane. (e) An unattached filament is bent under the membrane and exert entropic force *f*_*d*_.

Cell motility is essential for many biological processes including development, immune response, wound healing, phagocytosis and tumor metastasis. In order to crawl, many cells form a wide flat membrane protrusion, known as the lamellipodium, in the direction of movement. The main driving mechanism for lamellipodium formation is the force generated by treadmilling actin network underneath the membrane [1]. Actin treadmilling is a nonequilibrium process, requires the consumption of ATP [2] and is regulated by several proteins [3]. In this process, the barbed ends of actin filaments polymerize and push against the membrane, whereas their pointed ends are anchored in the gel bulk formed by strongly entangled and highly cross-linked actin filaments. The gel bulk is attached to the extracellular matrix via trans-membrane receptors providing a mechanical support for the weakly cross-linked actin brush at the leading edge to push against the membrane. A schematic presentation of the membrane, gel bulk and the actin brush in the so called semiflexible region, is shown in Fig 1.

In the semiflexible region, actin filaments perform thermal fluctuations and exert entropic force on the membrane [4]. These entropic forces are estimated to be strong enough to drive the membrane protrusion. Many proteins are known to act as permanent or transient cross-linkers between actin filaments, which provides mechanical stability to the network [5]. This cross-linking process keeps the free fluctuating length of the filaments in the semiflexible region short enough (∼ 100 nm) to exert larger entropic forces on the membrane. Thus, filaments are shortened by the cross-linking process and are elongated by a force-dependent polymerization rate; stronger the entropic force, lower the growth rate of the filaments. Moreover, there are experimental evidence that during the nucleation process in the presence of Arp2/3 complex, actin filaments can transiently attach to the membrane with a detachment rate that depends on the local force between the filament and the membrane. Membrane motion feeds-back onto the detachment and growth rate of actin filaments resulting in a rich coupled spatio-temporal dynamics of both the membrane and the underlying actin network.

In the gel region, the activity is powered by myosin II molecular motors. They bind to the actin filaments and upon ATP hydrolysis create internal stresses that contract the network [6]. These contractile forces pull the cytoskleton inward with respect to both the substrate and the cell boundary and are known as retrograde flow [7]. Consequently, the border between the semiflexible region and the gel bulk retracts due to actomyosin contraction and advances through the cross-linking process. Although the retrograde flow is an important aspect of cell movement, it can limit the efficiency of the protrusion. Experiments show that when the activity of myosin II is inhibited, the rate of retrograde flow decreases while the rate of membrane protrusion increases [7].

The plasma membrane is of central importance in the motility process. It defines the boundary separating the intracellular and extracellular environments, and mediates the interactions between a motile cell and its extracellular matrix. Furthermore, the membrane serves as a dynamic platform for localization of various components which actively participate in all aspects of the motility process, including force generation, adhesion, signaling, and regulation. As actin filaments polymerize and push against the cell membrane from within, membrane tension is build up. Forces on the membrane at any point equilibrate within milliseconds so that, on the time-scales relevant for motility, membrane tension is spatially homogeneous along the leading edge. The net protrusion rate of the membrane is determined by polymerization forces of actin filaments at the leading edge, retrograde flow driven by contraction of the actin gel, cell adhesion to the substrate and the membrane tension that imposes an opposing force on growing actin filaments.

A model that accounts for the dynamics of both actin filaments in the semifelxible region and the gel bulk was proposed in [8, 9] and extended in [10–14]. Two crucial assumptions of this model are as follows: first, in the vicinity of plasma membrane, the actin network is weakly cross-linked in the polymerization zone and highly cross-linked (gel-like) further back. Secondly, filaments in the semiflexible region can attach via linker proteins to the membrane, and detach with a force-dependent rate. Although the transition from a weakly cross-linked semiflexible region to a highly cross-linked gel bulk is gradual, for simplification purposes, it is described as a sharp transition and is called the gel boundary. In this model, the dynamics of filaments are described at the population level and the membrane is assumed to be under a constant tension that resists bending. This model offers quantitative explanations for steady motion and oscillation mechanisms in reconstituted systems and motile cells [10], formation of transient lamellipodium [15], membrane waves [12], and the force-velocity relation of fish keratocytes [16].

The goal of this study is to start with the simple version of the model introduced in Ref. [8] and describe the dynamics of membrane protrusion as a stochastic process. Note that the growth of actin filaments is indeed a stochastic process and the lengths of the filaments at any given time are likely to be unequal. In this toy model, we include explicitly dynamics of every single filament and consider stochasticity in attachment/detachment and growth processes. Furthermore, we model the membrane as a chain of beads and springs that evolve in the presence of forces exerted by the underlying actin network. Our aim is to investigate membrane dynamics as a function of various control parameters such as cross-linking velocity, retrograde flow driven by molecular motors and membrane tension. More specifically, we will focus on two scenarios: I) Myosin II-driven retrograde flow and cross-linking velocity compensate each other such that the net progression velocity of the gel boundary is negligible. II) Myosin II molecular motors are inhibited and as a result the retrograde flow is significantly reduced; In this scenario, dynamics of the gel boundary is dominated by the cross-linking velocity. Our simulations show that upon inhibition of molecular motors, the rate of membrane protrusion increases which is consistent with experimental observations reported in Ref. [7]. Our results are also consistent with the experimental observation that membrane elongation is inversely (linearly) proportional to the retrograde flow (cross-linking velocity); larger retrograde flows of the gel boundary “subtract” from cross-linking velocity and thereby long floppy filaments in the semiflexile region form that exert weaker forces on the membrane.

## Description of the model

The model we present here is the combination of series of events that are happening at the leading edge of the lamellipodium, and are influenced by the presence of plasma membrane. The system is made of three parts, the membrane, the gel boundary and the region confined between the gel boundary and the membrane, the so called semiflexible region (SR). Actin filaments in the SR can polymerize, depolymerize, attach to and detach from the membrane. These processes exert pushing or pulling forces to the point of contact of filaments with the membrane. While attached filaments are mostly under tension and pull back the membrane, detached filaments and the compressed attached filaments push the membrane forward. The free fluctuating tips of actin filaments are constantly polymerizing and depolymerizing with force dependent rates. They also attach to the membrane via linker proteins, with constant rate *k*_*a*_, and detach with force dependent rate *k*_*d*_ [14]. All filaments are firmly anchored in a cross-linked actin gel. The dynamics of the gel boundary is determined by retrograde flow and the cross-linking velocity. Retrograde flow is primarily generated by myosin contractile forces and depends on the strength of adhesion between the actin gel and extracellular matrix [17]. The gel is constantly formed by cross-linking process and acts as a mechanical support for the filaments in the SR to push against the membrane. Growing actin filaments push forward on the plasma membrane, resulting in membrane tension [18]. Forces on the membrane at any point equilibrate within milliseconds so that, on the time-scales relevant for motility, membrane tension is spatially homogeneous along the leading edge [19]. Moreover, membrane tension slows actin polymerization by pushing back on growing filaments [20–22]. When adhesion to the substrate is weak or absent, membrane tension pushing back on the filaments also generates retrograde flow of the actin network.

In the following parts, we first present our model for membrane dynamics in the presence of external forces. Then by characterizing the existing forces in the system, we define the equations for actin dynamics in the SR. Finally, we introduce the dynamics at the boundary of the gel and the SR.

### A. Membrane model

In this paper, we model the membrane by a chain of *N* + 1 beads connected by *N* springs with stretching modulus *k*_*m*_ [23, 24]. The effect of the adhesion to the substrate and surface tension of the membrane, are included in a force applied to the edges of the membrane [25], and these two edges are restricted to move only in the horizontal direction. The schematic picture of the model is presented in Fig 1(a), and a magnification of three beads on the membrane is shown in Fig 1(b). The discretized Helfrich energy for the deformed membrane can be written as

$$\begin{array}{c} G_{H}=\sum_{n=1}^{N}\frac{1}{2}\kappa_{m}a\frac{{\left[\Delta \left(\Delta {\vec{r}}_{n}\right)\right]}^{2}}{\Delta s_{n}^{3}}+\sum_{n=1}^{N}\frac{1}{2}k_{m}\left(\right|{\vec{r}}_{n}{\left|-a\right)}^{2}\;, \end{array}$$ (1)

where *κ*_*m*_ is the bending rigidity of the membrane [26], *a* is the equilibrium length of the springs, ${\vec{r}}_{n}$ is the vector connecting the bead *n* − 1 to the bead *n*, and $|{\vec{r}}_{n}|$ is its magnitude. In the above equation ${\left[\Delta \left(\Delta {\vec{r}}_{n}\right)\right]}^{2}$ and Δ*s*_*n*_ are defined as

$$\begin{array}{ccc} {\left[\Delta \left(\Delta {\vec{r}}_{n}\right)\right]}^{2} & \equiv & {\left(x_{n+1}+x_{n-1}-2x_{n}\right)}^{2}+{\left(y_{n+1}+y_{n-1}-2y_{n}\right)}^{2}\;, \end{array}$$ (2)

$$\begin{array}{ccc} \Delta s_{n} & \equiv & \frac{1}{2}[|{\vec{r}}_{n}|+|{\vec{r}}_{n+1}|]\;, \end{array}$$ (3)

where *x*_*n*_ and *y*_*n*_ are the Cartesian coordinates of the *n*-th bead. We note that *n*-th bead on the membrane can be under the influence of an external force, $\vec{F_{n}}$, as shown in Fig 1(b). This external force changes the free energy of the system as

$$\begin{array}{c} G_{F}=-\sum_{n=1}^{N+1}{\vec{F}}_{n}.{\vec{R}}_{n}\;, \end{array}$$ (4)

where the vector ${\vec{R}}_{n}$ is the displacement of the *n*-th bead under the influence of the external force. Now by combining the Helfrich energy, Eq (1), and the energy caused by the external force, Eq (4), we are able to calculate the membrane’s total energy as

$$\begin{array}{c} G_{tot}=G_{H}+G_{F}\;. \end{array}$$ (5)

For a given force distribution on the membrane, we use the membrane’s total energy *G*_*tot*_ to find the configuration of the membrane corresponding to the minimum energy. Since thermal fluctuations are unavoidable in biological systems, we allow each bead to slightly wiggle around its own minimum energy. A more detailed description of how the positions of beads are selected is presented in the supplementary materials. Finally, all of these steps are done several times to make sure that the membrane is relaxed before new sets of external forces are applied.

### B. Model of actin brush in semiflexible region

In our model, the actin brush consists of 25 actin filaments, with their pointed ends anchored inside the gel and their barbed ends vertically coming out of the gel. Actin filaments use the gel as a mechanical base to exert force on the membrane [14]. We emphasize that although in real cells density distribution of pushing actin filaments along the leading edge gradually decreases towards the cell side (with a maximum at the center) [21], throughout our simulations, we assume a sharp transition of filament density in SR.

The barbed end of each filament is free to fluctuate, polymerize and depolymerize in the SR [11]. In this system, if there is enough space available at the tip, a monomer of actin can get attached with a rate denoted by *k*_*p*_, while it can get detached from the tip with a rate *k*_*dp*_. In each case, the filament’s length changes by a monomer size, *δ*. Since both the membrane and the filament can fluctuate due to the thermal effects, a sufficient space can be provided between the tip of the filament and the membrane [27]. In addition, the filaments can also bind to the membrane temporarily via linker proteins with the rate *k*_*a*_. Fig 1(c) and 1(d) show the schematic configuration of the filaments when they are attached to the membrane. The linker protein is considered as a spring with a spring constant *k*_*l*_ and a negligible length. Furthermore, the filament is modeled as a spring with effective spring constant *k*_*f*_ defined as [28]

$$\begin{array}{c} k_{f}^{-1}=\frac{l_{p}^{2}}{k_{B}T}[\frac{2l}{3l_{p}}+\frac{2}{9}\left(e^{-\frac{3l}{l_{p}}}-1\right)-{\left(e^{-\frac{l}{l_{p}}}-1\right)}^{2}]\; \end{array}$$ (6)

where *l* is the filament’s contour length and *l*_*p*_ is the persistence length [26]. Attached filaments can either exert a pushing force when compressed (Fig 1(d)) or a pulling force when stretched out (Fig 1(c)). The pushing force is different from the force of detached filaments since the tip of the attached filament is not freely fluctuating. Force of attached filaments, denoted by *f*_*a*_, depends on several parameters such as (1) the linker protein spring constant, *k*_*l*_, (2) the linear response coefficient of a single attached filament, *k*_*f*_, (3) the contour length of the filament *l*, and (4) the distance between the anchoring point of the actin and the point where the force is acting on the membrane, *d*, and can be described as [12]

$$\begin{array}{c} f_{a}=\{\begin{array}{cc} -k_{f}\left(d-R_{e}\right), & \;d\leq R_{e}\\ -k_{eff}\left(d-R_{e}\right), & \;R_{e}<d\leq l\\ -k_{l}\left(d-l\right)-k_{eff}\left(l-R_{e}\right), & \;d>l \end{array} \end{array}$$ (7)

where *R*_*e*_ = *l*[1 − *l*/4*l*_*p*_] is the end-to-end distance of the filament, and *k*_*eff*_ is the effective spring constant of the linker-filament complex. Since the linker and the filament can be considered as two springs placed in series, the effective spring constant of the complex can be written as *k*_*eff*_ = *k*_*l*_ *k*_*f*_/(*k*_*l*_ + *k*_*f*_). As mentioned above, attached filaments can be compressed or stretched, depending on *d* and its contour length *l*. Considering this force, the detachment rate of the filaments from the membrane is given by

$$\begin{array}{c} k_{d}=k_{d}^{0}\;\exp[-\frac{f_{a}\delta }{k_{B}T}], \end{array}$$ (8)

where $k_{d}^{0}$ is the detachment rate in the absence of any forces [29].

An unattached actin filament in the SR can exert an entropic force on the membrane. This force is denoted by *f*_*d*_ and has been discussed extensively in Ref. [4]. It can be presented as $f_{d}=f_{c}{\tilde{f}}_{d}\left(\zeta \right)$, where *f*_*c*_ = *π*^2^*k*_*B*_*Tl*_*p*_/4*l*^2^ is the Euler buckling force and ${\tilde{f}}_{d}\left(\zeta \right)$ is given by

$$\begin{array}{c} {\tilde{f}}_{d}\left(\zeta \right)=\frac{4\exp(-\frac{1}{4\zeta })}{\pi^{5/2}\zeta^{3/2}[1-2\mathrm{erf}(\frac{1}{2\sqrt{\zeta }})]}, \end{array}$$ (9)

where erf denotes the error function and the dimensionless *ζ* is defined as *ζ* = *l*_*p*_(*l* − *z*)/*l*^2^. Here *z* is the normal distance between the anchoring point of the actin and the tangent line to the membrane at the position where the force is exerted [4]. Fig 1(e) show the configuration of an unattached filament that is bent and work against the membrane. Furthermore, detached filaments can polymerize at sub-second timescales and this growth process is influenced by the presence of membrane. The probability of monomer attachment decreases with increasing pushing force *f*_*d*_. The force-dependent polymerization and depolymerization rates of detached actin filaments are given by

$$\begin{array}{ccc} k_{p} & = & k_{p}^{0}\exp[-\gamma \frac{f_{d}\delta }{k_{B}T}], \end{array}$$ (10)

$$\begin{array}{ccc} k_{dp} & = & k_{dp}^{max}\exp[-\left(\gamma -1\right)\frac{f_{d}\delta }{k_{B}T}], \end{array}$$ (11)

where *γ* is the load distribution factor [30, 31] (assumed 0.5 in our simulations), $k_{p}^{0}$ and $k_{dp}^{max}$ are the polymerization and depolymerization rates in the absence of any external force, respectively. Moreover, $k_{p}^{0}$ depends on the distance Δ*h* between F-actin’s tip and the membrane and can be written as

$$\begin{array}{c} k_{p}^{0}=\{\begin{array}{cc} k_{p}^{max}, & \Delta h\geq \delta \\ k_{p}^{max}\exp\left(\frac{\Delta h-\delta }{\delta }\right), & \Delta h<\delta \end{array} \end{array}$$ (12)

with *δ* to be the radius of G-actin monomer and $k_{p}^{max}$ is the maximum polymerization rate.

Now, using Eqs (8), (10) and (11) and depending on the status of each actin filament (attached or unattached), the evolution rates are calculated. For the attached filaments the only available rate is the force-dependent detachment rate, while for the free fluctuating actin filaments we have the attachment, polymerization and depolymerization rates. We calculate a time span for each filament using these rates as

$$\begin{array}{c} dt^{\left(j\right)}=\{\begin{array}{cc} \frac{1}{k_{a}^{\left(j\right)}+k_{p}^{\left(j\right)}+k_{dp}^{\left(j\right)}}\;, & \text{unattached}\;\text{filament}\\ \frac{1}{k_{d}^{\left(j\right)}}, & \text{attached}\;\text{filament} \end{array} \end{array}$$ (13)

where the superscript *j* = 1, 2, …, 25 is the filament count in the system. Depending on the filament status, only some of the mentioned evolution processes are applicable. For instance, for an attached filament, the only possibility is detachment from the membrane. Therefore, the time needed for this filament to detach is equal to the reverse of *k*_*a*_. For an unattached filament, there are three possibilities as polymerization, depolymerization, and attachment. Hence, the total rate for possible activities of the filament is the sum over all of the mentioned rates. Next, we select the smallest *dt*^(*j*)^ among the calculated values and call it *dt*_*min*_. The state of each filament can change in the time interval *dt*_*min*_ according to its present state. An attached filament will be either detached from the membrane or remained attached. The probability of the detachment of filament number (*j*) from the membrane during time *dt*_*min*_ is $p_{d}^{\left(j\right)}=dt_{min}\times k_{d}^{\left(j\right)}$, where *k*_*d*_ is given by Eq (8). Therefore for each attached filament, after drawing a random number 0 ≤ *α* < 1, a decision is made as follows

$$\begin{array}{c} \text{attached}\;\text{filament}:\{\begin{array}{cc} 0\leq \alpha <p_{d}^{\left(j\right)}, & \;\;\text{filament}\;\text{detaches},\\ \alpha \geq p_{d}^{\left(j\right)}, & \;\;\text{nothing}\;\text{happens}. \end{array} \end{array}$$ (14)

For an unattached filament, there are four possibilities: (1) polymerization, (2) depolymerization, (3) attachment to the membrane, and (4) nothing happens. The probability of the mentioned possibilities for filament (*j*) during time *dt*_*min*_ are (1) $p_{p}^{\left(j\right)}=dt_{min}\times k_{p}^{\left(j\right)}$ corresponds to the polymerization, (2) $p_{dp}^{\left(j\right)}=dt_{min}\times k_{dp}^{\left(j\right)}$, and (3) $p_{a}^{\left(j\right)}=dt_{min}\times k_{a}^{\left(j\right)}$ corresponds to the attachment of the filament to the membrane. Then for each unattached filament, using the random number *α*, a decision is made as follows

$$\begin{array}{c} \text{unattached}\;\text{filament}:\{\begin{array}{cc} 0\leq \alpha <p_{p}^{\left(j\right)}, & \;\text{polymerization},\\ p_{p}^{\left(j\right)}\leq \alpha <p_{p}^{\left(j\right)}+p_{dp}^{\left(j\right)}, & \;\text{depolymerization},\\ p_{p}^{\left(j\right)}+p_{dp}^{\left(j\right)}\leq \alpha <p_{p}^{\left(j\right)}+p_{dp}^{\left(j\right)}+p_{a}^{\left(j\right)}, & \;\text{attachment},\\ \alpha \geq p_{p}^{\left(j\right)}+p_{dp}^{\left(j\right)}+p_{a}^{\left(j\right)}, & \;\text{nothing}\;\text{happens.} \end{array} \end{array}$$ (15)

It is worth mentioning that after the polymerization, the length of the filament increases by a monomer size *δ* and after the depolymerization the length of the filament decreases by *δ*. We note that because of the way of choosing *dt*_*min*_, for each time step and each filament the condition $dt_{min}\times \left(k_{a}^{\left(j\right)}+k_{p}^{\left(j\right)}+k_{d}p^{\left(j\right)}\right)\leq 1$ is satisfied.

Now having the proper model for the membrane dynamics and the actin filaments in the SR, we can calculate the forces that actin filaments exert on the membrane using *f*_*a*_ as defined in Eq 7, and $f_{d}=f_{c}{\tilde{f}}_{d}$ where ${\tilde{f}}_{d}$ is defined in Eq 9. The sum of these two forces for each filament replaces the external force *F*_*n*_ in the Eq (4), considering the fact that each actin filament applies a force normal to the membrane at the point of contact. As the membrane response is four orders of magnitude faster than any other time scales relevant for F-actin dynamics [19], the membrane relaxes under the influence of the applied forces instantly.

### C. Dynamics of the gel boundary

The mechanical properties of cells crucially depend on the physical properties of actin cortex, which is a thin, crosslinked actin network lying immediately beneath the plasma membrane [32]. Myosin motors exert contractile forces in the meshwork. Actin filaments are entangled via cross-linker proteins having a finite bound time. The treadmilling phenomenon and the action of myosin motors, however, introduce fundamentally novel aspects to the system [32, 33]. Gel bulk can attach via trans-membrane proteins to the substrate and thereby can play the role of a mechanical base for the filaments in the SR to push against the membrane. The gel boundary retracts due to the myosin-driven retrograde flow and advances via cross-linking process. Retrograde flow depends on the activity of molecular motors and also depends on the local forces exerted by filaments in SR on the gel boundary, and can be written as [12]

$$\begin{array}{c} v\left(l,f\right)=v_{g}\left(l\right)-v_{r}\left(f\right), \end{array}$$ (16)

where *v*_*g*_(*l*) denotes the cross-linking velocity, and *v*_*r*_(*f*) shows the retrograde flow. Depending on the filament status, *f* is equal to *f*_*a*_ or *f*_*d*_ for attached and detached filaments, respectively. The actin cross-linkers in the system are constantly dissociating from the gel bulk, diffusing forward and rebinding to the filaments in the SR, which gives us the length-dependent gel formation velocity as

$$\begin{array}{c} v_{g}\left(l\right)=v_{g}^{max}\tanh\left(l/\bar{l}\right), \end{array}$$ (17)

where $v_{g}^{max}$ is the maximum cross-linking/gel formation velocity, which depends on the concentration of the available cross-linkers in the length scale of $\bar{l}$, and *l* is the free length of the filament in the SR [11].

The other velocity which plays a crucial role in the system is the retrograde flow. It is caused by the constant transport of the gel from the leading edge to the back [34], and can be written as

$$\begin{array}{c} v_{r}\left(f\right)=a_{ret}^{0}+b_{ret}^{0}n_{a}f. \end{array}$$ (18)

Here the first retrograde flow term $a_{ret}^{0}$ is proportional to the active contractile stress and expresses retrograde flow arising from contraction in the actin network, e.g. due to myosin motor activity. The second term is proportional to the filament force *f* and filament density *n*_*a*_ and represents the actin network being pushed backwards due to week adhesion to the substrate. Note that *f* = *f*_*d*_ for a detached filament and *f* = *f*_*a*_ for attached filament. Therefore *f* is calculated for each single filament and is not an averaged quantity. Other important factors influencing retrograde flow are the viscosity of the actin gel, the height of the lamillipodium at the gel boundary and the length of the gel part of the lamellipodium [12, 17, 35]. In our simulations in the regime of week retrograde flow, e.g. due to the inhibition of molecular motors, we set $a_{ret}^{0}$ to zero. We use this expression of retrograde flow and Eq (17) for cross-linking velocity to calculate the displacement of the gel front, Δ*d*, as

$$\begin{array}{c} \Delta d=dt_{min}\;v\left(l,f\right), \end{array}$$ (19)

where *dt*_*min*_ denotes the time interval that has been discussed right after Eq (13), and *v*(*l*, *f*) has been introduced in Eq (16).

## Results and discussion

Combination of three sets of equations for membrane, gel boundary and actin filaments in the SR, as described above, breaks the initial flat configuration of the membrane and leads to the formation of a dynamic membrane protrusion. Our aim is to follow actin-driven spatio-temporal dynamics of the membrane and explore the effect of the gel dynamics on the membrane shape until a steady protrusion is established.

Based on experimental observations, we will investigate the membrane dynamics in two different regimes: I) retrograde flow compensates for the effect of the cross-linking process such that the net progression velocity of the gel boundary is negligible. We call this regime, “non-progressive” gel front. II) Upon inhibition of molecular motors [7], the retrograde flow is significantly reduced and the gel boundary advances by the cross-linking process. We call this regime “progressive” gel front, where the cross-linking velocity is significantly larger than the retrograde flow. Myosin inhibition reduces active contractile stress in the actin gel and can be simulated in our model by simply setting $a_{ret}^{0}=0$. Remarkably, we obtain longer membrane protrusions in the regime that the gel boundary is progressive. Our simulations show that membrane elongation is linearly proportional to the cross-linking velocity or equivalently, is inversely proportional to the retrograde flow; larger the retrograde flow, smaller the net progression velocity of the gel boundary and consequently smaller membrane protrusions.

In order to study the aforementioned regimes, progressive versus non-progressive gel boundary, we used the same set of parameters for membrane and actin filaments. Membrane consists of *N* = 700 connected springs which corresponds to a length of 7 *μm*. Initially, the membrane is considered as a completely straight line plus thermal fluctuations with a tension *F*_*e*_ applied to its edges. The initial width of SR is considered 150 *nm* and it contains 25 filaments, which are perpendicular to the gel front with density *n*_*a*_. The filaments initially are made of 55 actin monomers (*l* = 148.5 *nm*), therefore they are completely relaxed. The rest of parameters introduced in the paper and used in the model are presented in Table 1. In each step, the actin system evolves according to the force-dependent rates. Consequently, filaments exert new set of forces on the membrane and membrane movement feedbacks on the actin dynamics.

**Table 1:** List of parameters used in our simulations.

Parameter	Value
Stretching modulus, *k*_*m*_	227 *pNnm*^−1^ [23]
Membrane bending rigidity, *κ*_*m*_	20 *k*_*B*_*T* [26]
Thermal energy, *k*_*B*_*T*	4.14 *pNnm*
Equilibrium length of the springs, *a*	10 *nm* Assumed
G–actin monomer size, *δ*	2.7 *nm* [26]
Actin persistent length, *l*_*p*_	15 *μm* [26]
Saturation length of cross-linking velocity, $\bar{l}$	100 *nm* [11]
Linker proteins spring constant, *k*_*l*_	1.0 *pNnm*^−1^ [28]
Attachment rate, *k*_*a*_	1 *s*^−1^ [14]
Force-free detachment rate, $k_{d}^{0}$	0.5 *s*^−1^ [29]
Force-free depolymerization rate, $k_{dp}^{max}$	1.4 *s*^−1^ [36]
Filaments density, *n*_*a*_	0.1 *nm*^−1^ [37]
Retrograde flow coefficient, $a_{ret}^{0}$	0
Retrograde flow coefficient, $b_{ret}^{0}$	80 *nm*^2^ *pN*^−1^ *s*^−1^ [12]
Tension of the edges, *F*_*e*_	3 – 24 *pN* [25]
Maximum polymerization rate, $k_{p}^{max}$	35 – 60 *s*^−1^ [38]
Maximum gel formation velocity, $v_{g}^{max}$	10 – 40 *nms*^−1^ Assumed

### Balance between retrograde flow and cross-linking velocity

To investigate the effect of gel boundary on membrane dynamics, we first focus on the case that the net movement of the gel boundary is negligible. This corresponds to the case that retrograde flow and cross-linking velocity are balanced such that the gel boundary doesn’t move effectively (see S1 Video).

**Fig 2:**
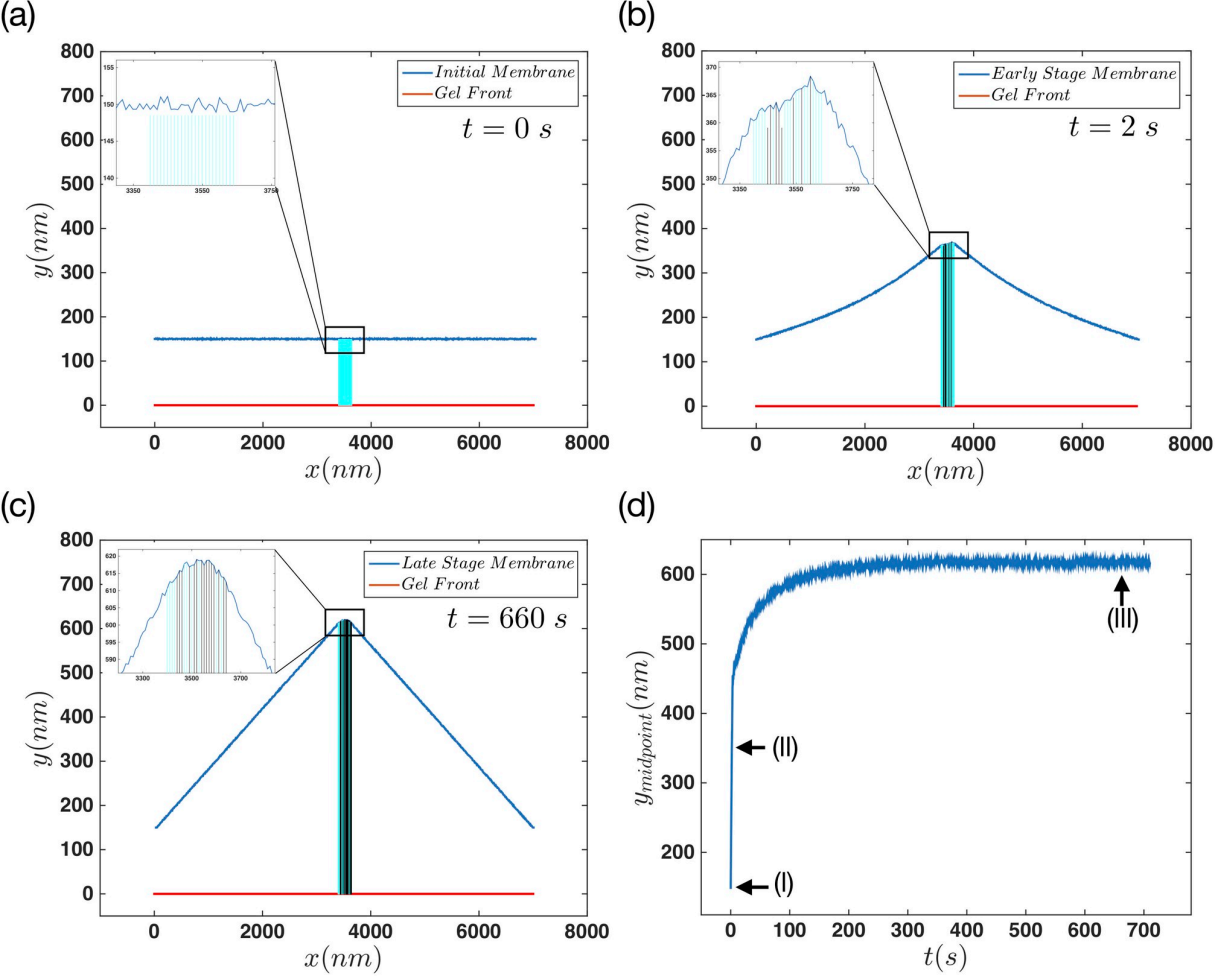
Membrane dynamics in the regime that cross-linking and retrogarde flow are balanced. (a) Initial (b) early and (c) late steady configuration of the membrane for *n*_*a*_ = 0.1 *nm*^−1^, *F*_*e*_ = 12 *pN*, $k_{p}^{max}=60\;s^{-1}$. The insets in panels (a)-(c) shows the filaments status under the membrane. The light-blue lines represent the free fluctuating filaments while the black line shows the filaments in their attached state. The red line shows the non-progressive gel front located at *y* = 0. (d) Time evolution of the middle point of the membrane and the little arrows show the points where the data for the (a)-(c) plots are collected.

Fig 2(a)–2(c) show the initial, early and late stages of the system after evolving for several thousands of steps, respectively. Fig 2(d) shows the time evolution of the middle point of the membrane. The three arrows in this panel show the points where the data for initial, early and late stages are retrieved from. Furthermore, Fig 2(d) show two stages of movement in this system, which we call early and late stages. In the first stage, in which the membrane moves forward quite fast, the length of filaments change quickly due to polymerization and depolymerization.

**Fig 3:**
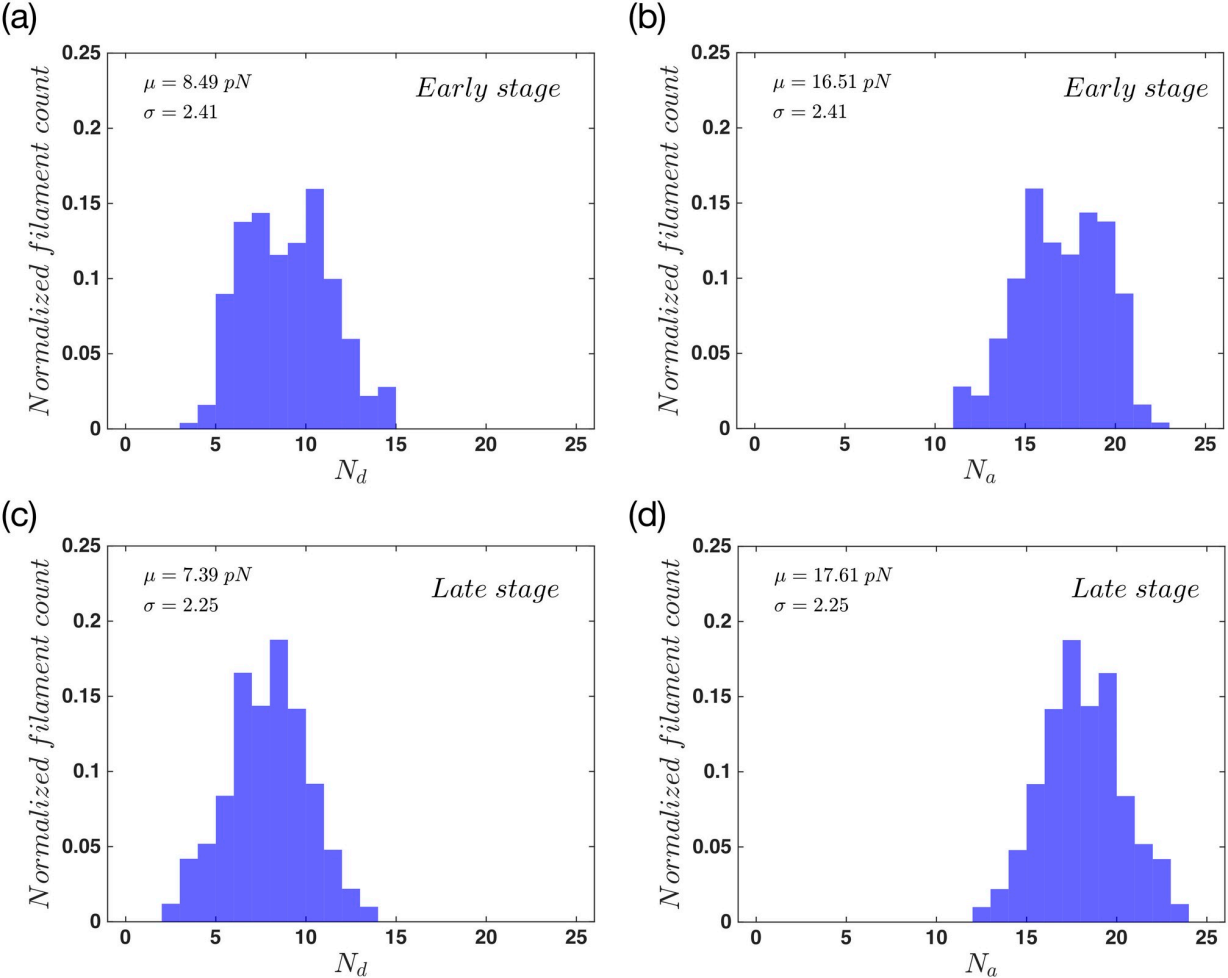
Filament distribution in the system with non-progressive gel boundary. The probability distribution of (a) detached and (b) attached filaments for the early state and also the probability distribution of (c) detached and (d) attached filaments for the late stage of the whole movement. This is the result for a system of *n*_*a*_ = 0.1 *nm*^−1^, *F*_*e*_ = 12 *pN* and $k_{p}^{max}=60\;s^{-1}$. The total number of filaments is constant and equal to 25 during the whole process. Here *μ* and *σ* are the mean value and standard deviation of a normal distribution fitted to the data.

As a filament attaches to the membrane via linker proteins, it stops growing or shrinking while the neighboring free filaments can still change their status. If these filaments grow and push the membrane forward, the detachment probability of the attached filament increases exponentially with force. Finally, the filament detaches from the membrane. Therefore in the early stage the constant attachment and detachment of filaments to the membrane is detected. During the late stage, the forces that filaments apply on the membrane are balanced out with membrane tension and bending and thereby, the membrane does not move any further. Fig 3(a) and 3(b) show the normalized number of attached and detached filaments during the early stage of the movement. To obtain these data we took 500 steps in the early stage, and counted the number of attached and detached filaments in each step. Then we used the whole number of states, to normalize the results. The results show a wide distribution for the number of attached (*N*_*a*_) and detached (*N*_*d*_) filaments. Fig 3(c) and 3(d) show the normalized distribution of the same parameters but this time for the late stage of the movement. In comparison to the early stage, here we find a slightly narrower distribution. As the system approaches to the late stage, the number of attached filaments in each step increases. This is due to the force dependency of filaments detachment rate, Eq (8). As the filaments get longer, and also due to the fact that they are confined to the SR, they are compressed under the membrane. Therefore the attachment force *f*_*a*_ becomes positive and the detachment rate decreases. This will result in an increase in the number of attached filaments, as we get closer to the late stages of the movement.

**Fig 4:**
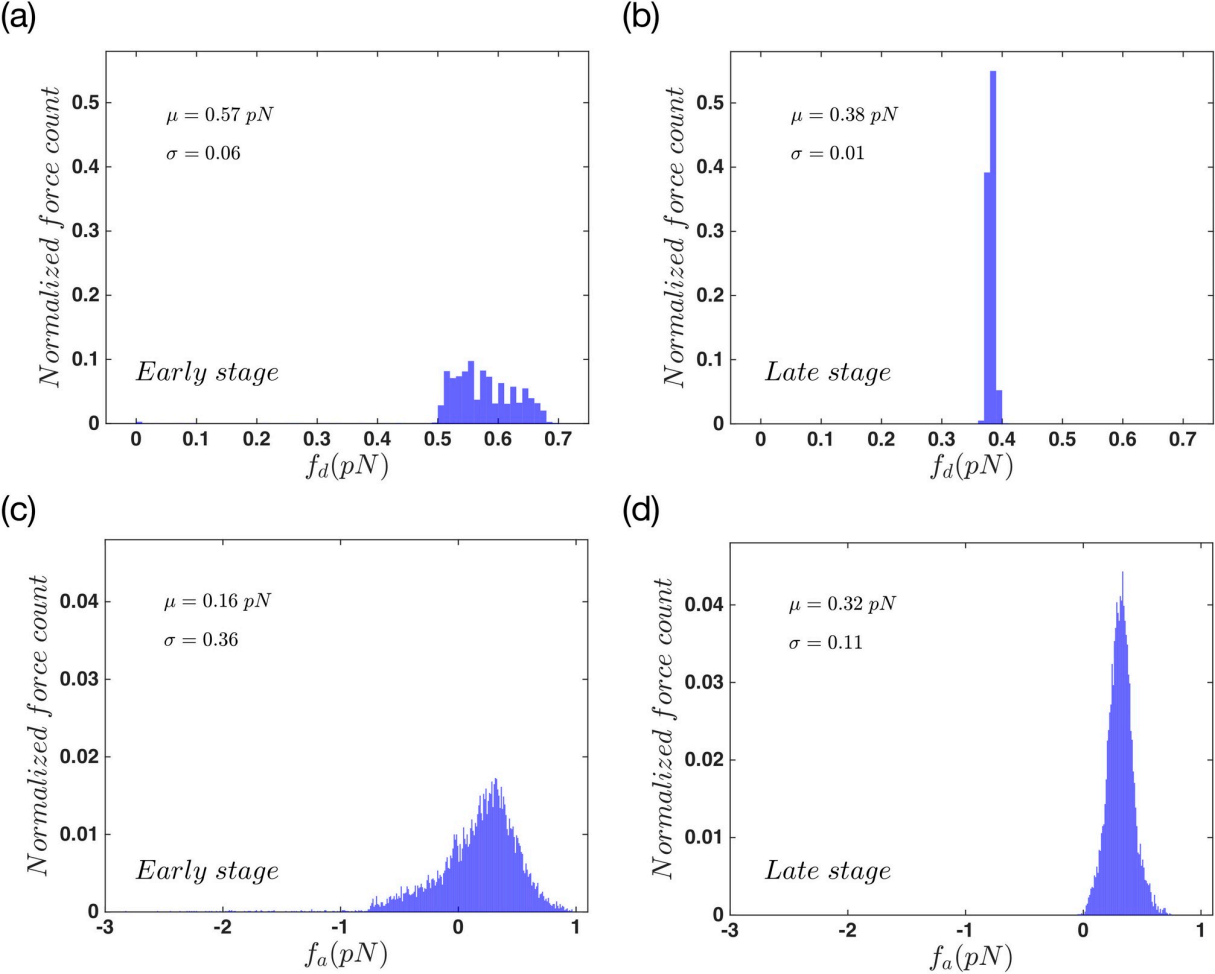
Force distribution in the system with non-progressive gel front. Force distribution of detached actin filaments in (a) early stages, (b) late stages of the protrusion. The attached force distribution of actin in (c) early stages and (d) late stages of the total movement. Here *μ* and *σ* are mean value and standard deviation of a normal distribution fitted to the data.

To analyze the system dynamics in more details, we looked carefully at the forces that attached and detached filaments exert on the membrane. Fig 4(a) and 4(b) show the detachment forces at the early and the late stage of movement, respectively. During the early state, as the filaments have shorter lengths, the magnitude of the force they apply on the membrane is quite bigger, and it is also widely distributed. This is due to the fact that the scaling coefficient of the *f*_*d*_ is the Euler buckling force which is proportional to *l*^−2^. During the late stage, the force distribution becomes narrower with smaller magnitudes. By moving towards the late stage, filaments grow longer and are slightly compressed underneath the membrane and exert smaller forces on the membrane; see Eq (6) for the length dependency of the effective spring constant of filaments.

Fig 4(c) and 4(d) also show the normalized force distribution but this time for the attached status of filaments. Panel (c) presents a distribution of forces with a tail which spreads to negative magnitudes. This shows that in the early stage, part of attached filaments/linker proteins are being stretched out as the membrane moves forward. By looking at the late stages of attachment force distribution, in Fig 4(d), we again find a narrow distribution but with no negative tail. This happens because the length of attached filaments is such that they are completely compressed under the membrane. This force is balanced with membrane tension and bending, and is not pushing the membrane any further.

**Fig 5:**
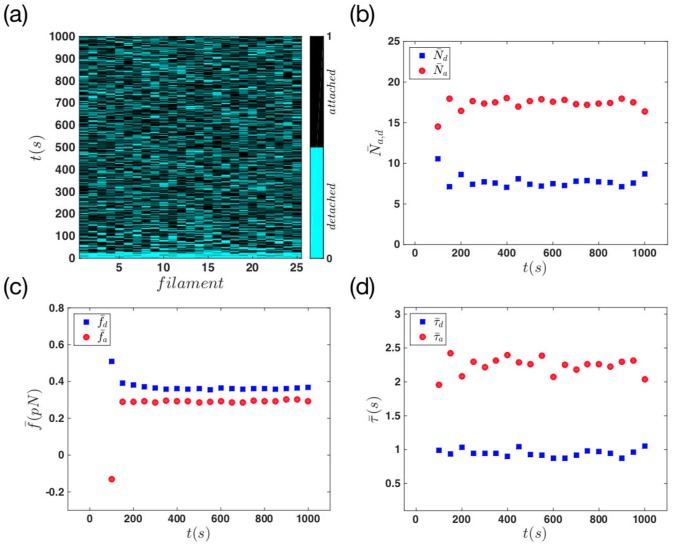
Mean quantities in the regime of non-progressive gel front. (a) Status of actin filaments during the whole simulation time. For this system the attachment rate is constant, *k*_*a*_ = 1 *s*^−1^, and the force-free detachment rate is $k_{d}^{0}=0.5\;s^{-1}$. We have 25 filaments in the system which are shown in the horizontal axis. The blue and the black color represent the detached and attached status of the filaments, respectively. (b) The mean number of attached and detached filaments versus time. (c) The average force that attachment and detachment filaments apply on the membrane as a function of time. (d) The mean attachment and detachment time of the filaments versus time.

Another quantity that we measured in our simulations, is the average attached and detached times, *τ*_*a*,*d*_. Fig 5(a) shows the color-coded state of the filaments during 1000 *s* of system evolution. In Fig 5(b) and 5(c) we calculated the average number of attached and detached filaments and the average attachment and detachment forces for every 50 *s* of the movement. Panel (d) shows the mean time *τ*_*a*,*d*_ which filaments spend in each of the attached and detached status, for the same time span. The results in panel (b) show a rapid increase (decrease) in the number of attached (detached) filaments as we move towards the later stages of the movement. As the system reaches the late stage, the mean number of attached and detached filaments stay almost constant.

A similar behavior is observed as we looked at the average forces in the system. In Fig 5(c), as the number of attached filaments increases, the mean attachment force increases to a constant value; This increase in the number of attached filaments, balances out the effect of force reduction caused by growth of filaments length. Therefore the mean force stays constant as the system reaches the late stages of the movement. Finally in panel (d) we measured constant mean attachment and detachment times. Here ${\bar{\tau }}_{d}$ is approximately equal to 1 *s* which means on average one filament is attaching to the membrane in every second. This is in agreement with constant attachment rate *k*_*a*_ = 1 *s*^−1^ assumed in our model. The mean attachment time, ${\bar{\tau }}_{a}$, of the system is constant and it is approximately equal to 2.2 *s*. If we extract the mean attachment force from panel (c) (or from the histogram in Fig 4(d)), and using the Eq 8, we obtain *τ*_*a*_ = 2.4 *s* which is in agreement with the value extracted from Fig 5(c).

### Weak retrograde flow and dominance of cross-linking velocity

**Fig 6:**
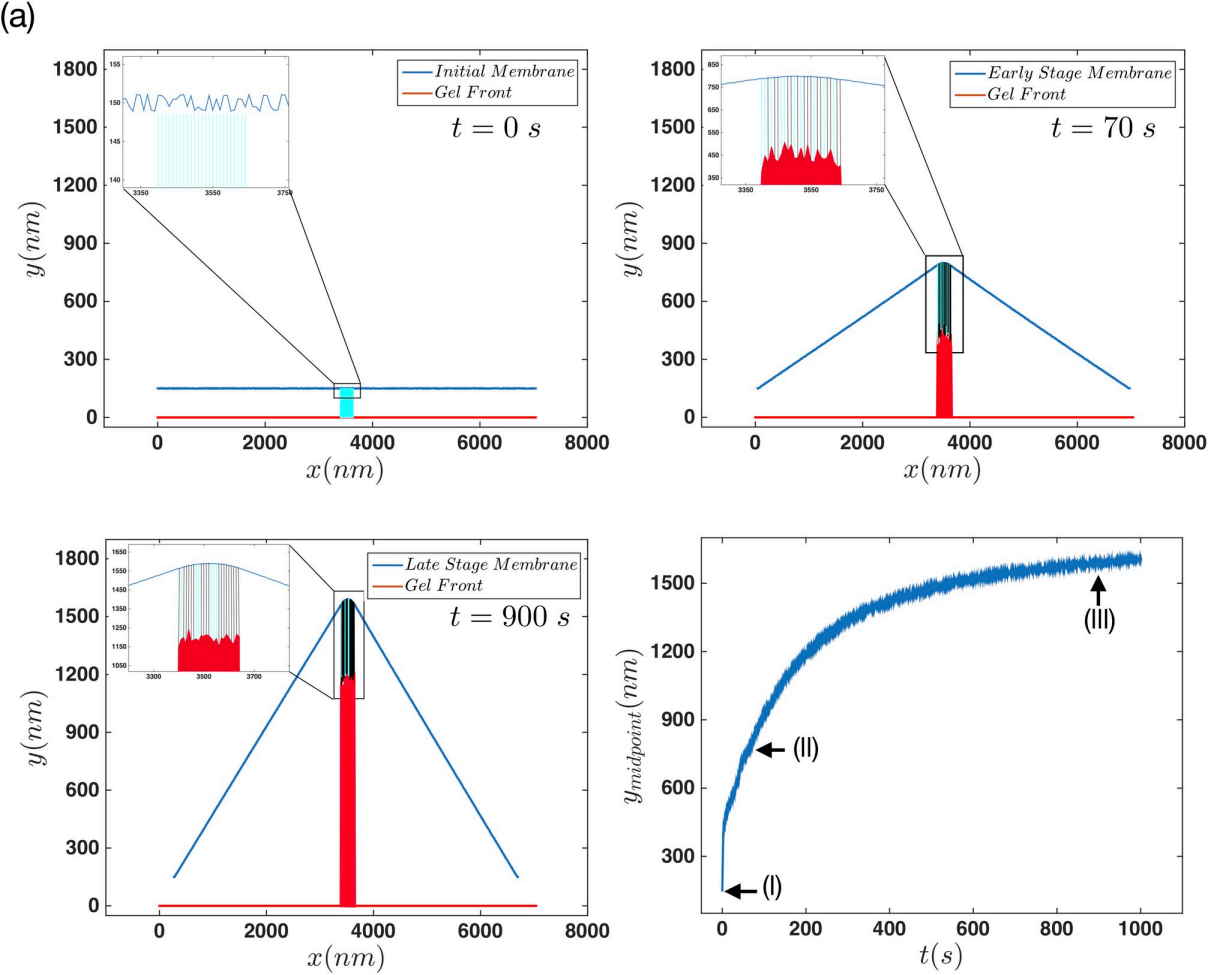
Membrane deformation in the regime of progressive gel. (a) Initial (b) early and (c) late configuration of the membrane and gel for *n*_*a*_ = 0.1 *nm*^−1^, *F*_*e*_ = 12 *pN*, $k_{p}^{max}=60\;s^{-1}$ and $v_{g}^{max}=10\;nms^{-1}$. The insets in panels (a)-(c) show the filaments status underneath the membrane. The blue lines represent the free fluctuating filaments while the black lines show the attached filaments. (d) Behavior of the middle-point of the membrane versus time. The little arrows show the points where the data for the (a)-(c) plots are collected.

Inhibition of molecular motors reduces the gel contractility and thereby retrograde flow is diminished. In this case, the important parameter that determines the dynamics of the gel boundary is the cross-linking velocity which together with other parameters such as maximum polymerization velocity and membrane tension determine the final shape of the membrane. Fig 6 and S2 Video show the behavior of the system in different stages of the protrusion.

Similar to the regime of non-progressive gel front, there are two stages of the membrane movement, early and late (see Fig 6). One important difference is the magnitude of the membrane protrusion which is quite larger in the progressive gel regime. Here besides filaments growing and pushing the membrane forward, the gel gets formed by shortening the length of free fluctuating filaments in the SR. Thus short filaments can exert stronger forces on the membrane which then result in larger elongation of the membrane.

**Fig 7:**
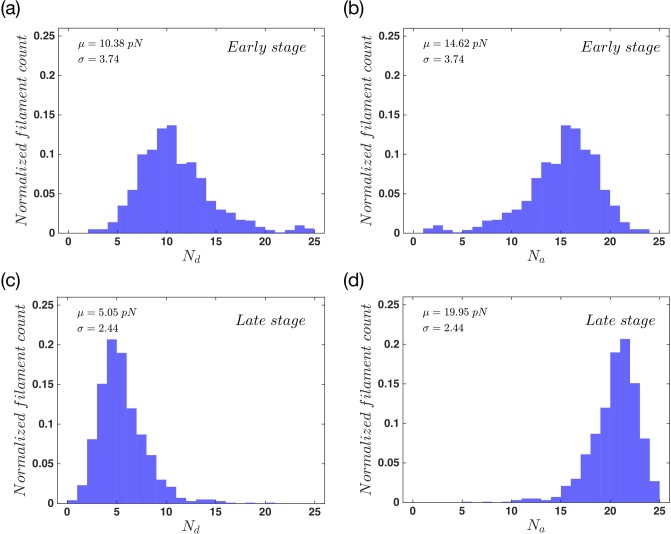
Filament distribution in the system with progressive gel front. The probability distribution of (a) detached and (b) attached filaments for the early state and (c, d) the late stage of membrane protrusion. The total number of filaments is constant and equal to 25 during the whole process. Here *μ* and *σ* are mean value and standard deviation of a normal distribution fitted to the data. Parameters are *n*_*a*_ = 0.1 *nm*^−1^, *F*_*e*_ = 12 *pN*, $k_{p}^{max}=60\;s^{-1}$ and $v_{g}^{max}=10\;nms^{-1}$.

We also examined the normalized number of attached and detached filaments during the early and late stages of the membrane protrusion. Fig 7(a)–7(d) show the probability distribution of actin filaments in both stages of the protrusion. Here we observe the same behavior as the non-progressive gel case. During the early stage we have a wide distribution while during the late stages this distribution gets quite narrower. It also shifts to having more attached filaments in each step as we move towards the late stage. The difference here is the mean number of attached filaments in the late stage, which is slightly bigger for the regime of progressive gel boundary.

**Fig 8:**
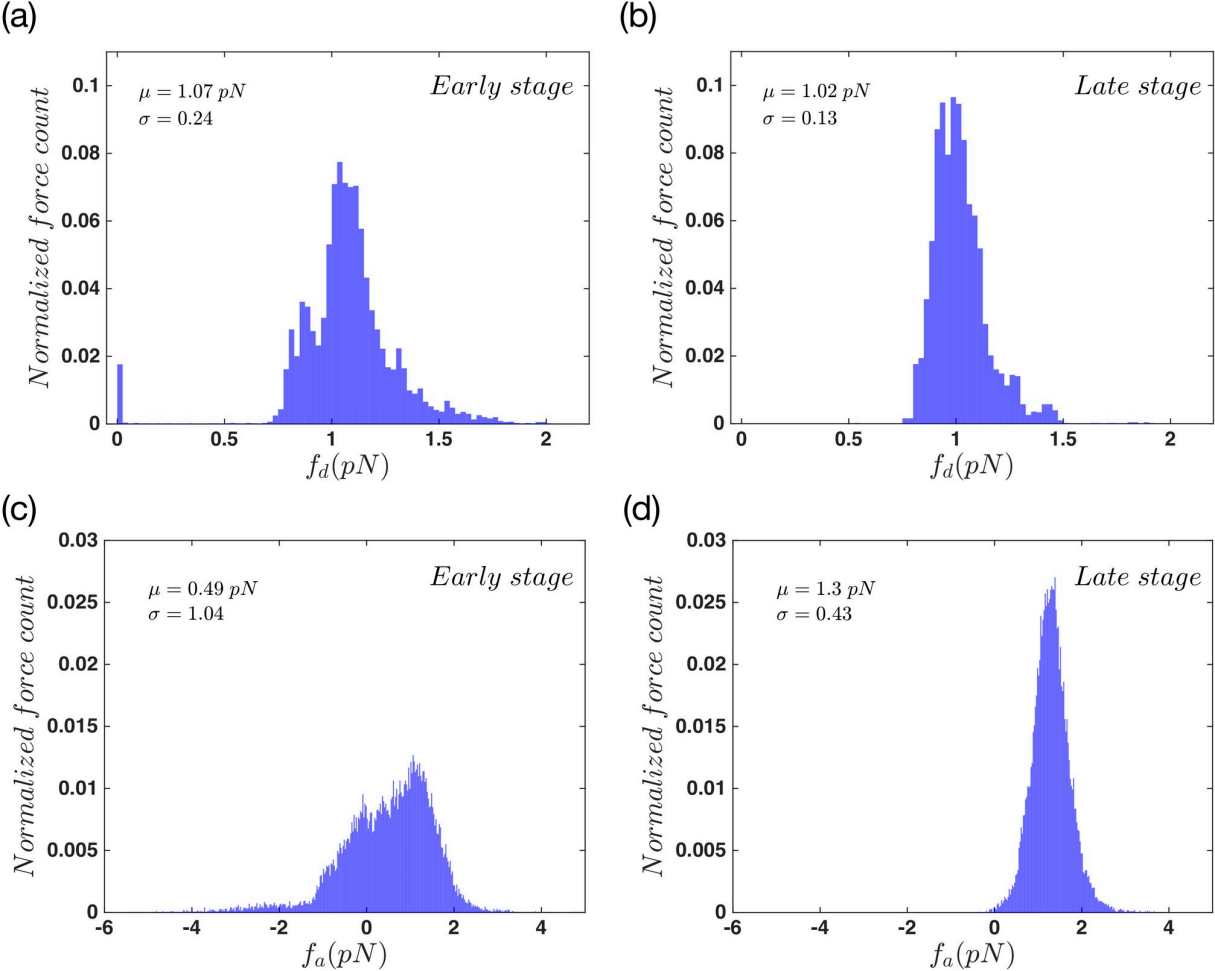
Force distribution in the system with advancing gel. Force distribution of actin filaments in detached status of (a) early stages, (b) late stages of the protrusion. The attached force distribution of actin in (c) early stages and (d) late stages of the total movement. The gel part in this system is active. Here *μ* and *σ* are mean value and standard deviation of a Normal distribution fitted to the data.

To study this phenomena we looked carefully at the pushing and pulling forces of attached and detached filaments. Fig 8(a) shows the probability distribution of detached forces in the early stage and Fig 8(b) shows the same data in the late stage of the protrusion. These plots show an opposite behavior in comparison to the non-progressive case. During the early state the detached force distribution is wide, but it stays wide in the bigger forces as we reach the late stage. Also the force magnitude gets bigger in the late stage. Due to the cross-linking process the length of filaments shrink as the gel boundary moves forward in space. Therefore there exist a wide distribution of actin length in the system and more importantly bigger forces as we get to the late stage. There also exist a large number of cases where the filaments apply a very small force on the membrane. This means that a number of filaments are quit relax under the membrane. This behavior is the result of gel activity. Fig 8(c) and 8(d) show the normalized distribution of *f*_*a*_ in the aforementioned states. Here we get a wide distribution that spreads towards the positive and negative values. It keeps its shape during the whole period of movement but it gets slightly narrower as it reaches the late stage. In both cases of *f*_*a*_ and *f*_*d*_, the magnitude of the forces are bigger than the non-progressive gel front. This is because a portion of filament length is incorporated into the gel during cross-linking process.

**Fig 9:**
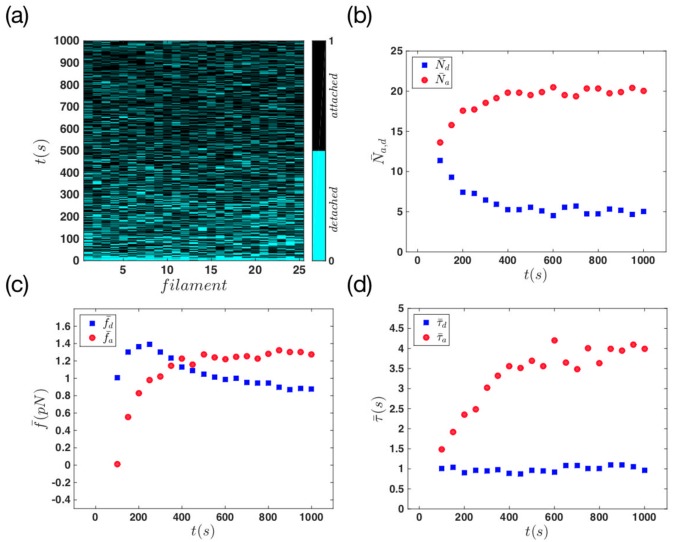
Mean quantities for the case of progressive gel front. (a) Actin filaments status during the whole evolution for the case of active gel. We have 25 filaments in the system which are shown in the horizontal axis. The blue color shows the detached status of the filament and the black shows the attached status. (b) The average force exerted by attached and detached filaments on the membrane as a function of simulation time. (c) The mean attachment and detachment time of the filaments versus time.

Now we take a look at the average attached and detached times, *τ*_*a*,*d*_ in the regime of progressive gel front. Fig 9(a) shows the status of the filaments during 1000 *s* of simulation. This plot shows an increase in the attached status of filaments in the course of time. Fig 9(b) shows an increase in the number of attached filaments in the system, which agrees with the results shown in panel (a). This increase happens with two different slopes. It is quite fast at the early phase of movement and saturates at the late stage. In panel (c) we show the mean attachment and detachment forces that filaments exert on the membrane. Here the attachment force increases in the same manner as ${\bar{N}}_{a}$, while a different behavior is detected for ${\bar{f}}_{d}$. During the early stage, the detachment force increases to a certain point and then it enters a constant decreasing phase. This phenomena is due to the fact that in the initial state, all filaments are in the relaxed condition and as long as there is no contact between them and the membrane, they exert no force or rather a very small force on the membrane. The single isolated peak around *f*_*d*_ = 0 in Fig 8(a) shows the same effect, which continues to the early stage. As the system moves forward and the tension of the membrane increases, more and more filaments get involved in the force exertion process. But again due to the fact that the mean number of detached filaments is decreasing and also the filaments are getting longer, the over all force they apply on the membrane starts to decrease.

Finally, we looked at the mean attachment and detachment times of the system in Fig 9(d). The mean detachment time, ${\bar{\tau }}_{d}$, does not change with time. This behavior is expected as the attachment rate of the filament is force independent and it is equal to *k*_*a*_ = 1 *s*^−1^. But as the rate of detachment depends exponentially on the attachment force, we expect ${\bar{\tau }}_{a}$ to increase as the ${\bar{f}}_{a}$ is increasing. The results shown in panel (c) agrees with our expectation. Similar to non-progressive gel scenario, we extract the force from panel Fig 9(c) (or Fig 8(d)), ${\bar{\tau }}_{a}=4.6\;s$ is calculated which agrees with the data shown in panel Fig 9(d).

**Fig 10:**
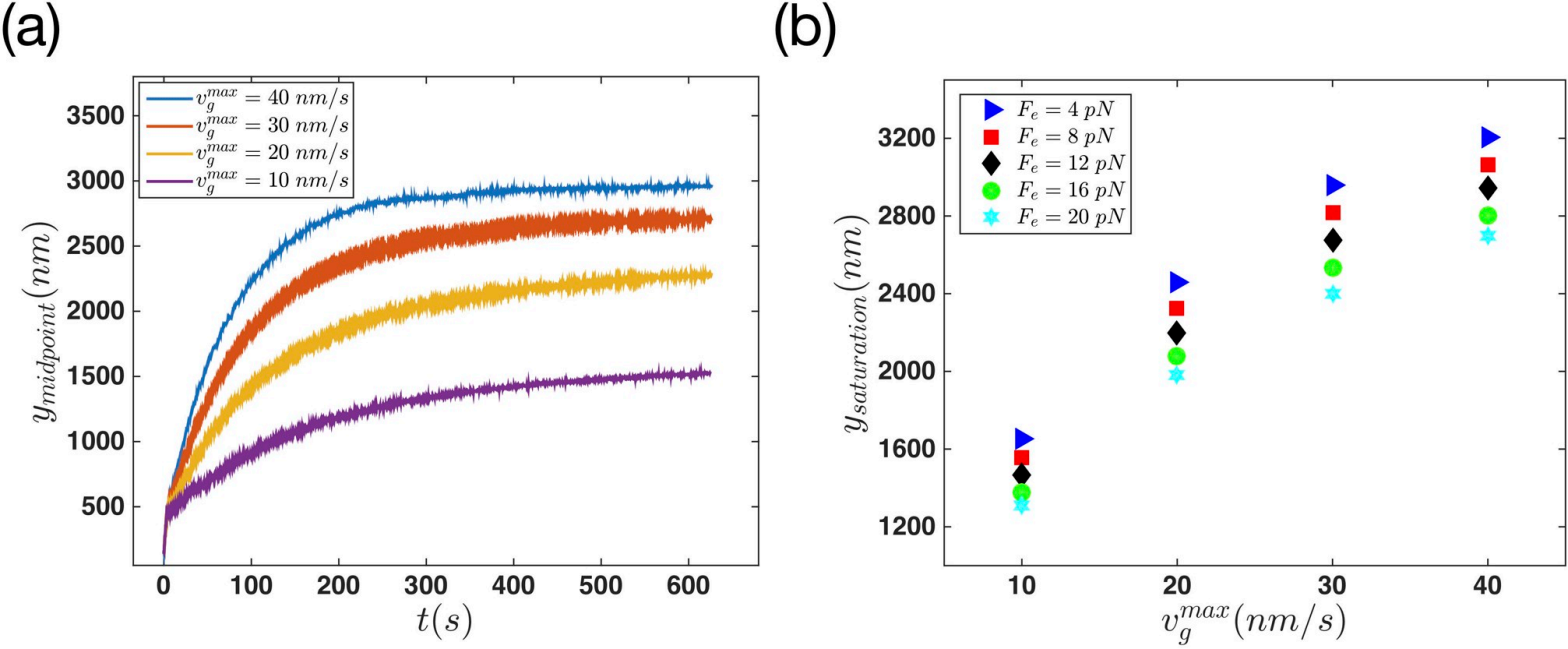
Effect of gel cross-linking velocity on membrane protrusion. (a) The dynamics of membrane’s middle point versus time for different values of $v_{g}^{max}=\left[10,20,30,40\right]\;nm/s$. In this case the force *F*_*e*_ = 12 *pN* is applied to the edges, and the filaments polymerization rates is equal to $k_{p}^{max}=52\;s^{-1}$. (b) Maximum elongation of the membrane versus $v_{g}^{max}$ at various values of *F*_*e*_.

One important parameter influencing the magnitude of the protrusion is the gel cross-linking velocity, $v_{g}^{max}$. Fig 10 shows the behavior of the membrane’s middle point when the gel formation velocity changes from 10 *nms*^−1^ to 40 *nms*^−1^. It is quite considerable that this parameter plays a crucial role in the formation and dynamics of the protrusion in the cell body. As the gel formation velocity grows, the protrusion in the membrane becomes bigger and the membrane elongates more. In the cross-linking process, gel constantly decreases the free length of filaments in the SR. This phenomena increases the magnitude of the force filaments are applying on the membrane. Therefore the same number of filaments here, are able to increase the tension in the membrane more than the regime where the gel front was frozen. By a small change in the cross-linking velocity, the magnitude of protrusion changes quite considerably.

**Fig 11:**
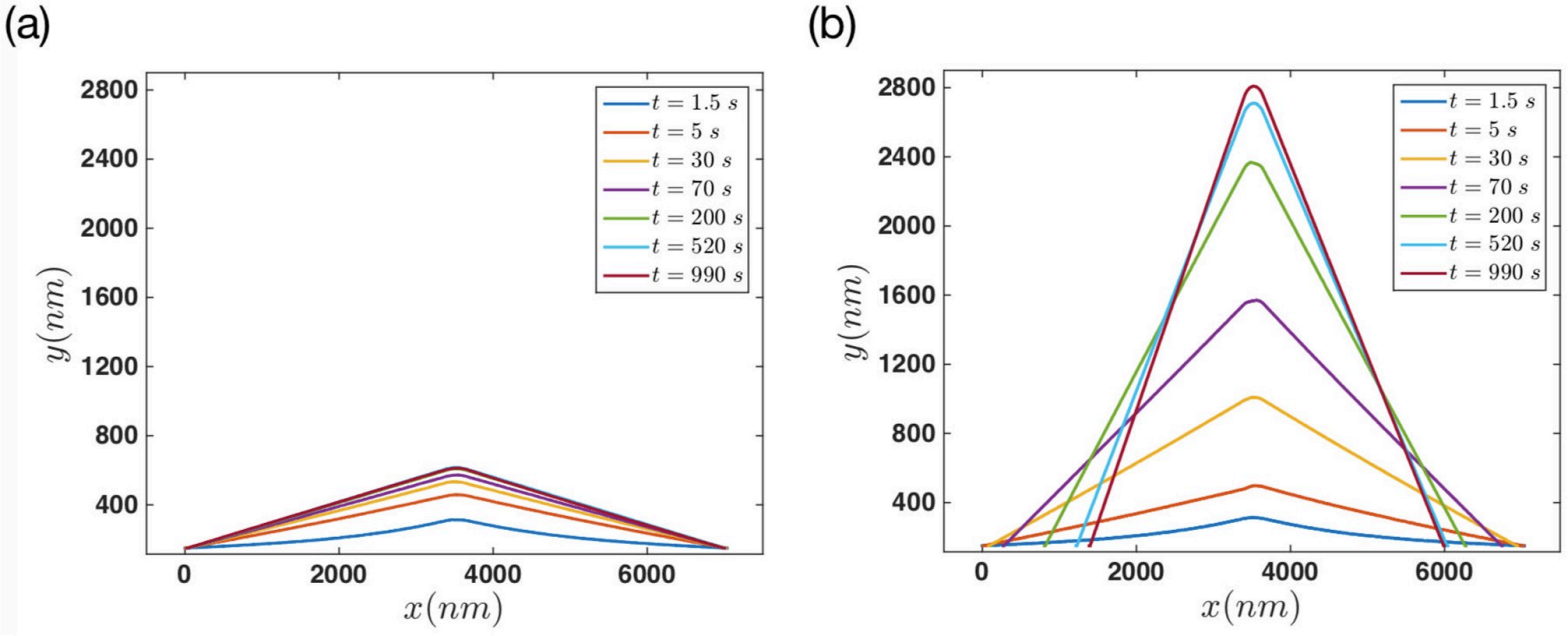
Comparison of membrane deformations between progressive versus non-progressive gel boundary. (a) In the case of non-progressive gel front, the final steady configuration is established much earlier and the final membrane elongation is smaller than a progressive gel front. Here $k_{p}^{max}=60\;s^{-1}$, *F*_*e*_ = 12 *pN* and $v_{g}^{max}=30\;nm/s$ for progressive gel scenario.

To compare our simulation results for two different regimes of progressive versus non-progressive gel boundary, we looked at the membrane’s deformation at different time points. Fig 11(a) shows the membrane configuration at various times for the case that the gel front is non-progressive. Panel (b) shows the membrane at the same time points after adding activity to the gel front. It is interesting to observe a steady behavior for the non-progressive gel boundary after 200 seconds. While in the case of progressing gel front filaments push the membrane quite further in the same time span.

**Fig 12:**
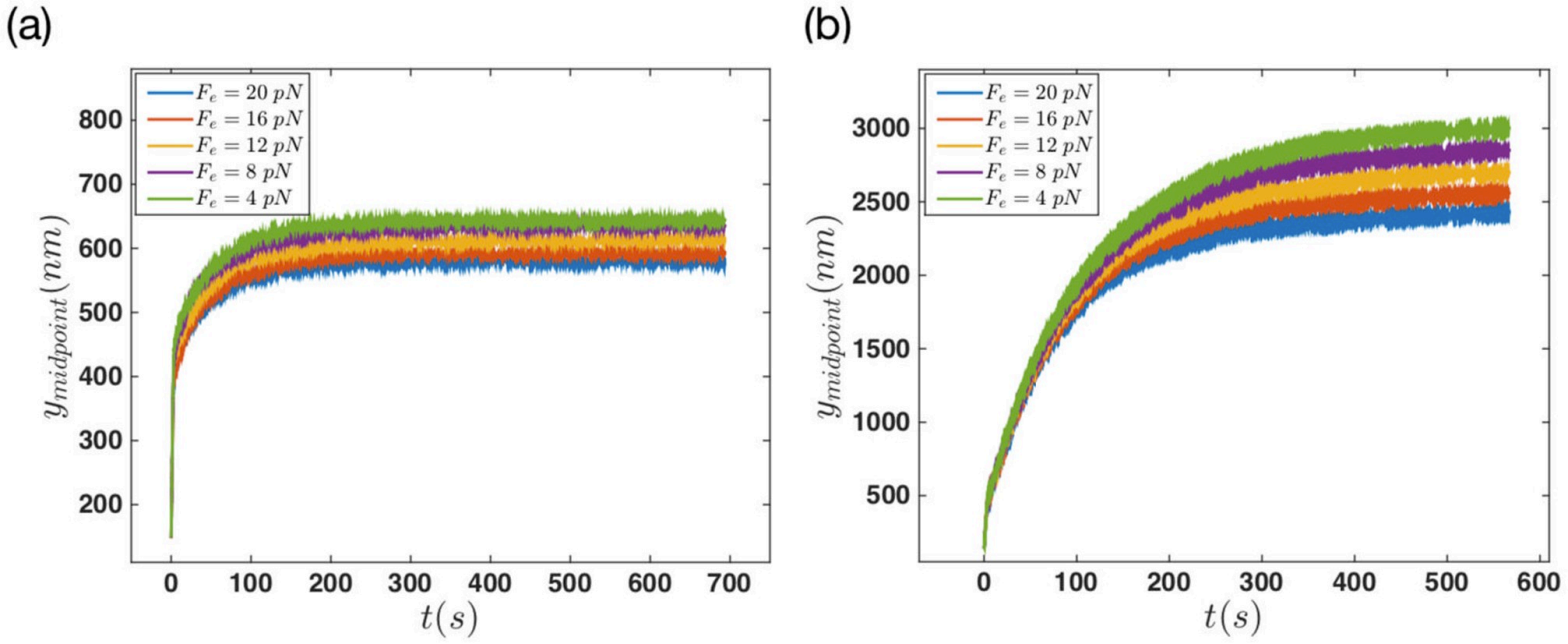
Final steady state configuration of the membrane is reached faster in the case of non-progressive gel for different values of edge tension. The dynamics of membrane’s middle point versus time for different values of edge tension *F*_*e*_ = [4, 8, 12, 16, 20] *pN*, with filaments polymerization rate equal to $k_{p}^{max}=52\;s^{-1}$. (a) Non-progressive versus (b) progressive gel boundary with $v_{g}^{max}=30\;nm/s$.

Finally, we looked at the effect of two other control parameters, namely, edge tension and maximum polymerization velocity on membrane protrusion. Fig 12 shows the behavior of the membrane’s middle point plotted against time when various tensions are applied to the edges. Fig 12(a) and 12(b) show the results of the non-progressive and progressive gel boundary, respectively. In both systems the effect of edge tension is quite obvious. The membrane protrudes longer as the edge tension is decreased. Interestingly, this effect is more significant in the regime of progressive gel boundary. Note that the membrane stretching modulus *k*_*m*_ (see Eq 1) can also be considered as a parameter to tune membrane stiffness. S4 Fig shows the membrane deformation with various values of *k*_*m*_ for both progressive and non-progressive gel boundary.

**Fig 13:**
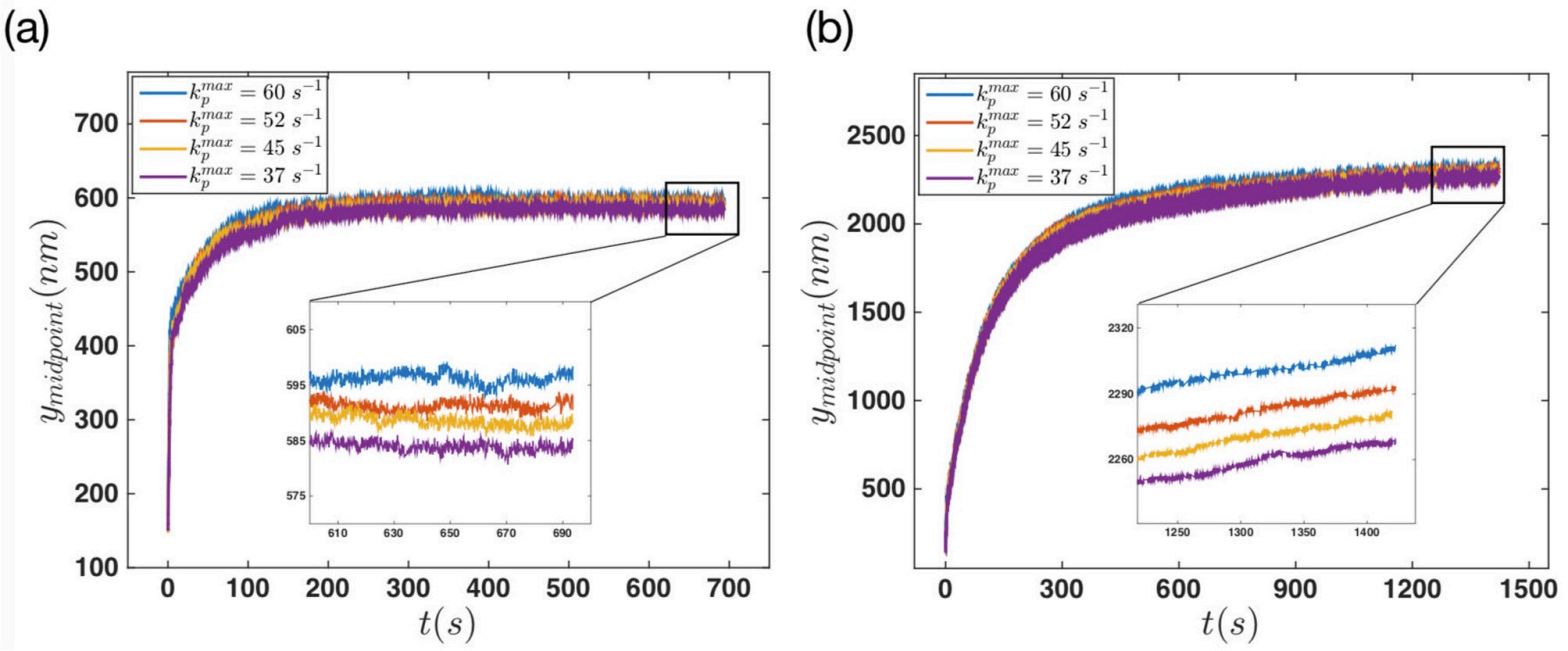
Final steady state configuration of the membrane is reached faster in the case of non-progressive gel for different values of polymerization rate. The dynamics of membrane’s middle point versus time for different values of $k_{p}^{max}=\left[37,45,52,60\right]\;s^{-1}$, with the force *F*_*e*_ = 16 *pN* applied to the edges. (a) Non-progressive gel versus (b) progressive gel boundary with $v_{g}^{max}=20\;nm/s$.

The time trace of the membrane’s middle point for different values of the maximum polymerization rate is shown in Fig 13. The results show a small variation in the magnitude of final membrane protrusion as the polymerization rate changes from $k_{p}^{max}=37\;s^{-1}$ to $k_{p}^{max}=60\;s^{-1}$. This small increase can be seen in both non-progressive and progressive gel boundary, which is only of the order of few tens of nanometers.

## Conclusion

A lamellipodium is a flat and broad membrane extension filled with a dense and highly cross-linked filament network. Force generation through actin polymerization has been believed to be the essential driving mechanism in formation of membrane protrusion. The main purpose of this article is to understand how the presence of resistive restoring force of the plasma membrane affects force generation of polymerizing actin filaments and membrane protrusion dynamics. We modeled the membrane as a series of beads connected by springs which deform in the presence of protrusive forces of the underlying actin network. We coupled explicitly stochastic attachment/detachment and growth processes of actin filaments with fluctuation dynamics of the plasma membrane to investigate membrane protrusion as a function of various control parameters such as cross-linking velocity, retrograde flow driven by molecular motors and membrane tension.

A key outcome of our simulations is that membrane protrusion dynamics depends sensitively on the activity of the gel front which is defined as the boundary between the gel bulk and the semiflexible region. We specifically examined the role of myosin-driven retrograde flow that retracts the gel boundary, and cross-linking process that causes the gel boundary to advance. Combination of these two processes determine the net progression velocity of the gel boundary which directly influences the free fluctuating length of the filaments in the SR, and thereby affect the membrane dynamics. In the regime that retrograde flow and cross-linking velocity are balanced, the gel boundary doesn’t advance effectively and the resulting membrane elongation is much smaller than the regime that retrograde flow is significantly reduced. Since a large part of the retrograde flow is driven by gel contraction, upon inhibition of myosin II molecular motors, the retrograde flow becomes much smaller than cross-linking velocity and the gel boundary advances towards the membrane by reducing the contour length of the filaments in SR. These short filaments are more effective (than long filaments in the first regime) on exerting strong entropic forces on the membrane which leads to the formation of long membrane protrusions. Below, we summarize our main findings:

- The time scale for the formation of stationary lamellipodium in the case of progressive gel boundary is slower, compared to the non-progressive gel scenario, but the final membrane elongation is larger.
- Membrane elongation is linearly proportional to the cross-linking velocity at the gel boundary or equivalently, is inversely proportional to the retrograde flow.
- The mean attachment time of the filaments to the membrane is larger in the case of progressive gel boundary.
- The mean protrusive force of actin filaments is larger in the case of progressive gel boundary compared to non-progressive one.
- The mean number of attached filaments increases in both progressive and non-progressive regimes as the system moves towards the stationary state.
- Among tested parameters, $v_{g}^{max}$, *F*_*e*_ and $k_{p}^{max}$, the maximum velocity of gel formation was the most effective parameter in enhancing membrane protrusion. The force applied to the edges of membrane, *F*_*e*_, was the second most effective parameter.

**Fig 14:**
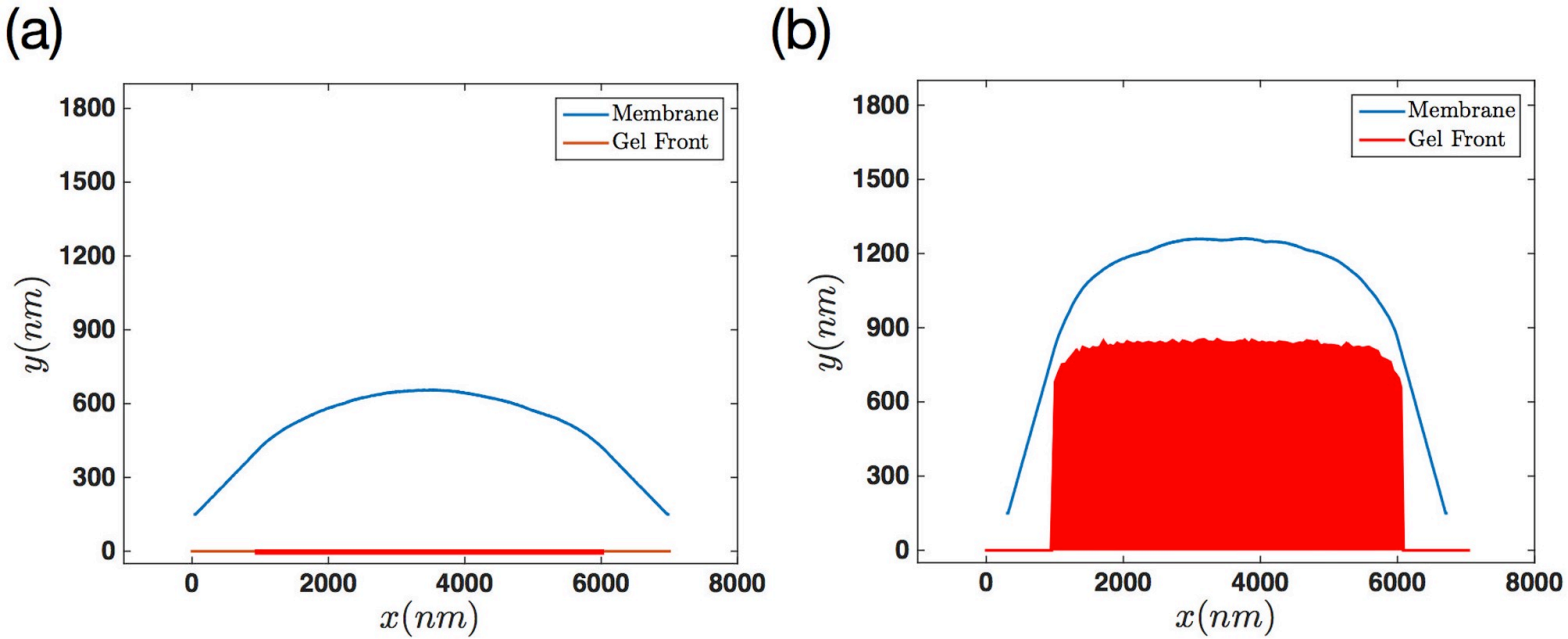
Snapshots of membrane’s protrusion with a wider actin brush underneath the membrane. Upon distribution of actin filaments on a larger region, we obtain lamellipodium-like protrusions for (a) non-progressive and (b) progressive gel boundary. Total number of filaments is 101 and the parameters are $k_{p}^{max}=60\;s^{-1}$, *F*_*e*_ = 12 *pN*, $v_{g}^{max}=30\;nm/s$ and *n*_*a*_ = 0.02 *nm*^−1^.

Throughout our simulations, we have assumed a narrow region with a sharp transition for filaments in SR that push against the membrane. As a result, we obtain tent pole like membrane protrusions, as shown in Fig 11. However, we emphasize that the final steady state shape of the membrane depends on the system parameters. Lamellipodium-like protrusions can be achieved in our simulations by changing these parameters. We performed numerical simulations for both active and passive gel front with the modification that hundred filaments (instead of 25) are distributed over a wider length (5 *μ*m instead of 0.25 *μ*m) on the gel boundary. These filaments are spaced 50 nm away from each other (instead of 10 nm) which is equivalent to reducing the density from 0.1 *nm*^−1^ to 0.02 *nm*^−1^. Fig 14 shows exemplary membrane deformations in these type of simulations with a wider actin brush underneath the membrane.

Finally, in this study, we have not discussed the processes of nucleating new actin filaments via Arp2/3 complex or capping of the already existing ones via capping proteins. While in our model the overall number of actin filaments is constant, in the real cells nucleation/capping processes can change the filament density underneath the membrane. Such cellular processes create short filaments that can exert strong entropic forces on the membrane, or remove long filaments that are not effective in pushing against the membrane. These events are expected to influence the dynamics of membrane protrusion and we aim to incorporate them into our model. Furthermore, the model presented for the gel dynamics simplifies the viscoelastic dynamics of the gel by just focusing on the gel boundary. We plan to derive the dynamical equations of the gel boundary from the viscoelsatic properties of the active gel and investigate the effects on membrane deformations.

## Supporting information

S1 File Energy minimization. (PDF)

S1 Fig Three bead energy minimization in absence of external force. (a) Three arbitrary initial positions for the beads plus two configurations with minimum energy of the middle bead. (b) Energy landscape of the system plus the minimum energy position marked with dark and light blue.
(TIFF)

S2 FigThree bead energy minimization with external force.(a) Three arbitrary initial positions with force equal to $\vec{f}=30\hat{j}\;pN$ applied to the middle bead plus the configuration of the global minimum energy of the system. (b) 3D energy landscape of the system due to probing the space with the middle bead. The projected contour lines show the position of the global minimum in the system.(TIFF)

S3 Fig Thermal fluctuation ring. 2D energy landscape of three bead system containing marked possible areas (inside red rings) for thermal fluctuation.
(TIFF)

S4 FigThe effect of changing the membrane stretching modulus on the size of protrusion.The dynamics of membrane’s middle point versus time for different values of membrane stretching modulus *k*_*m*_ = [41, 83, 228, 455] *pN*/*nm*, with filaments polymerization rate equal to $k_{p}^{max}=52\;s^{-1}$ and the edge force *F*_*e*_ = 12 *pN*. (a) Non-progressive versus (b) progressive gel boundary with $v_{g}^{max}=30\;nm/s$.(TIFF)

S1 VideoProtrusion formation in passive gel case.Video of actin polymerization and the membrane response while the gel part is passive. Here the *F*_*e*_ = 12 *pN* and $k_{p}^{max}=45\;s^{-1}$. The upper frame shows the whole system while the lower is a zoomed presentation of what happens at the tip of protrusion.(MOV)

S2 VideoProtrusion formation in active gel case.Video of actin polymerization and the membrane response in presence of the active gel. Here the *F*_*e*_ = 12 *pN*, $k_{p}^{max}=45\;s^{-1}$ and $v_{g}^{max}=10\;nm/s$. The upper frame shows the whole system while the lower is a zoomed presentation of what happens at the tip of membrane and gel protrusion.(MOV)
