# Supplementary material for: Influence of cross-linking and retrograde flow on formation and dynamics of lamellipodium
Source: PLoS One. 2019 Mar 21;14(3):e0213810. doi: 10.1371/journal.pone.0213810 (PMC6428246; doi:10.1371/journal.pone.0213810)
Supplement: S1 File — (PDF) [file pone.0213810.s001.pdf]

Supplementary Information

Membrane Relaxation

For energy minimization, we start from the middle point on the membrane and move our way towards the edges. Each time, we take three neighboring points, which is schematically has been shown in Fig 1 (b). In this configuration the two points on the edges are fixed while the point in the middle can easily move. Depending on the external force being applied to the middle point or not, we have two cases: (1) absence, and (2) presence of external force.

In the absence of external force- In order to find the configuration corresponding to the minimum energy, we make a fine grid over the shaded area of Fig 1 (b) and probe the energy of the system by moving the middle point on the grid and calculating the energy using the following equation

$$G_{H,n} = \frac{1}{2}\kappa_m a \frac{[\Delta(\Delta\vec{r}_n)]^2}{\Delta s_n^3} + \sum_{i=0}^{1} \frac{1}{2} k_m (|\vec{r}_{n+i}| - a)^2, \tag{1}$$

where $G_{H,n}$ is the Helfrich energy corresponding to the n-th bead.

S1 Fig (a) shows an arbitrary initial position for the three points and also the final position after energy minimization. S1 Fig (b) shows the energy landscape of the shaded area in the system and the colored points show the position of the minimum of energy in the system. In order to minimize the energy we used the parameters presented in Table 1.

Due to the fact that there exist two curvatures which give us the same energy, we can find at most two configurations correspond to the minimum energy. The position of these points are marked with light and dark blue on the energy landscape of S1 Fig (b). As both of these configurations are equally likely to happen in the system, we choose randomly between them.

In the presence of external force- Our second case is the time where there is an external force present. We go through the same procedure plus adding the effect of external force while calculation of the energy,

$$G_{F,n} = -\vec{F}_n \cdot \vec{R}_n, \tag{2}$$

where $G_{F,n}$ denotes the energy changes due to the presence of force F acting on the n-th bead.

This time the outcome is slightly different. Now the external force causes a shift in the energy landscape, making one of the configurations the global minimum and the other one a local. Therefore, in the presence of external force, we have just one minimum of energy and move the middle point to that coordinates. S2 Fig shows the configuration of the points and energy landscape when an external force equal to $\vec{f} = 30\hat{\jmath}\ pN$ is applied to the middle point. Here $\hat{\jmath}$ is the normal unit vector in y direction. This choice of force is just due to getting a better visual sense of the local and global minimum in the system.

Now after each energy minimization, we move to the neighboring points towards the edges of the ribbon. After evolving all points on the membrane, we should update the external forces. As mentioned in the actin system model in SR subsection, the force that an actin filament exerts on the membrane, depends on the configuration of filaments beneath it. Now that the membrane has relaxed to a new position, the filament applies a different force on the membrane and therefore the external forces should be modified. We repeat the aforementioned processes of membrane relaxation and external force modification for several times.

After finding the membrane relaxed configuration, we let each point fluctuate around the point with minimum value of energy. We use the same aforementioned mechanism with a slight difference. For each three points on the membrane we find the energy of the middle piece and we let it jiggle around this energy as a result of thermal fluctuation. First we make a fine grid, this time in the area closer to the points with minimum of energy. Later we find all the points which their energy is located in the rang of $[G_{min}, G_{min} + k_B T]$.

S3 Fig shows the same energy landscape of S1 Fig (b) with the specific fluctuation area bound with the red rings. Now we count the number of points found in this area and by generation of a random number with a white noise distribution, we randomly pick one point. This selected position will give us the final configuration of our three point system. We apply this method to each and every point on the membrane.

Various membrane stretching modulus effect on protrusion

We have also studied the effect of changing the membrane stretching modulus on the formation of the protrusion. S4 Fig (a) and (b) show this results for the non-progressive and progressive gel scenarios. It is clear that by increasing the membrane stretching modulus the magnitude of the protrusion decreases in both cases.

Supplementary Figures

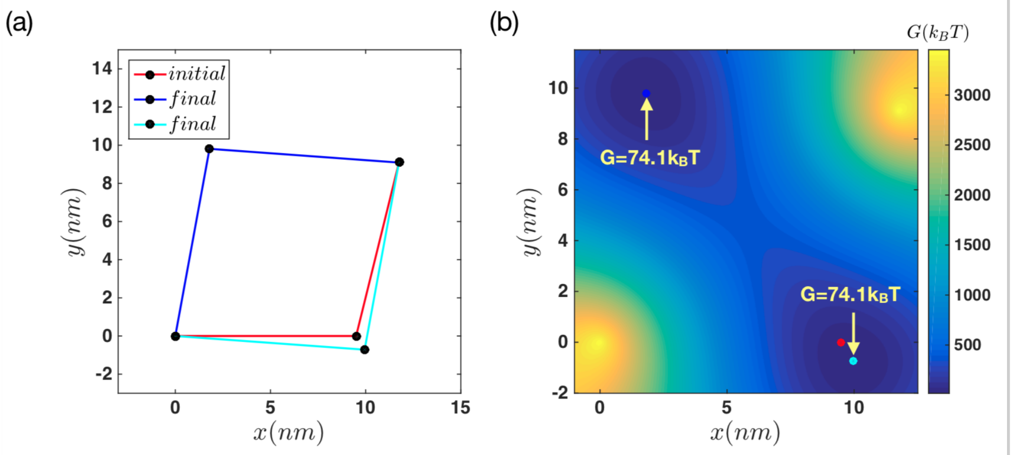

Fig S 1. (a) Three arbitrary initial positions for the beads plus two configurations with minimum energy of the middle bead. (b) Energy landscape of the system plus the minimum energy position marked with dark and light blue.

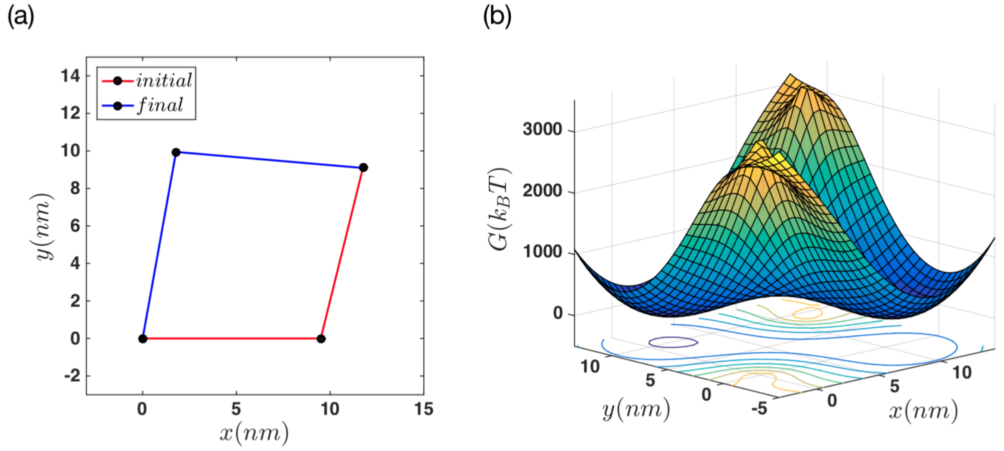

Fig S 2. (a) Three arbitrary initial positions with force equal to $\vec{f} = 30\hat{j}\ pN$ applied to the middle bead plus the configuration of the global minimum energy of the system. (b) 3D energy landscape of the system due to probing the space with the middle bead. The projected contour lines show the position of the global minimum in the system.

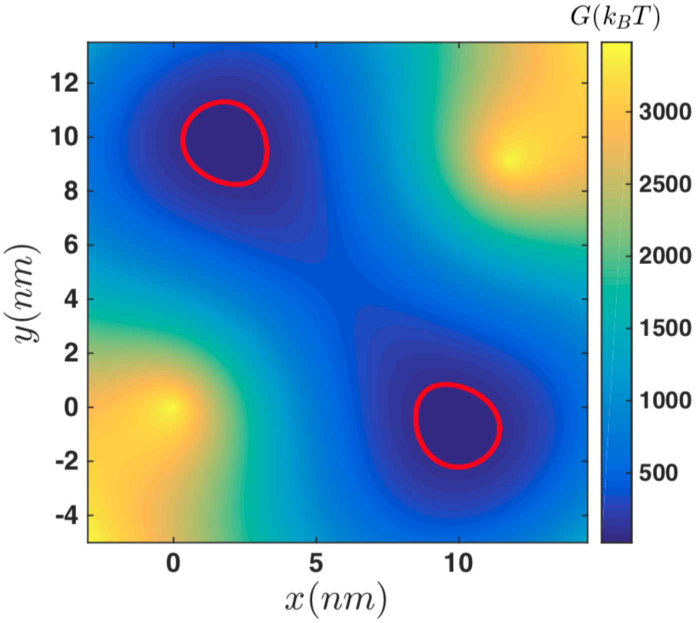

Fig S 3. 2D energy landscape of three bead system containing marked possible areas (inside red rings) for thermal fluctuation.

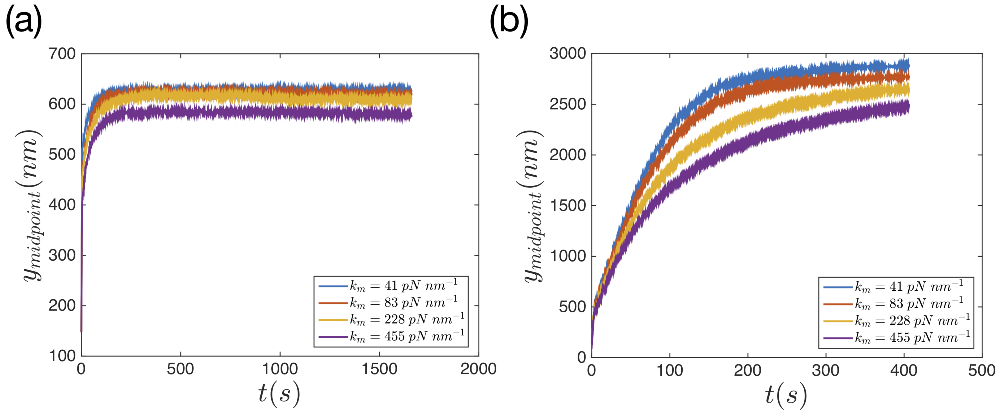

Fig S 4. The dynamics of membrane's middle point versus time for different values of membrane stretching modulus $k_m = [41, 83, 228, 455]\ pN/nm$, with filaments polymerization rate equal to $k_p^{max} = 52\ s^{-1}$ and the edge force $F_e = 12\ pN$. (a) Non-progressive versus (b) progressive gel boundary with $v_g^{max} = 30\ nm/s$.
